# Revealing new tick-borne encephalitis virus foci by screening antibodies in sheep milk

**DOI:** 10.1186/s13071-020-04030-4

**Published:** 2020-04-08

**Authors:** Amélie Wallenhammar, Richard Lindqvist, Naveed Asghar, Sezin Gunaltay, Hans Fredlund, Åke Davidsson, Sören Andersson, Anna K. Överby, Magnus Johansson

**Affiliations:** 1 School of Medical Sciences, Inflammatory Response and Infection Susceptibility Centre (iRiSC), Faculty of Medicine and Health, Örebro University, Örebro, Sweden; 2 Department of Clinical Microbiology, Virology, Laboratory for Molecular Infection Medicine Sweden (MIMS), Umeå University, Umeå, Sweden; 3 Present Address: Lydia Becker Institiute of Immunology and Inflammation, Faculty of Biology, Medicine and Health, Manchester Academic Health Science Centre, University of Manchester, Manchester, UK; 4 Department of Laboratory Medicine, Faculty of Medicine and Health, Örebro University, Örebro, Sweden

**Keywords:** Tick-borne encephalitis virus, *Flavivirus*, Tick-borne encephalitis, Hotspot, TBE virus foci, Sweden, Milk, Pasteurisation, Alimentary TBE virus transmission

## Abstract

**Background:**

Tick distribution in Sweden has increased in recent years, with the prevalence of ticks predicted to spread towards the northern parts of the country, thus increasing the risk of tick-borne zoonoses in new regions. Tick-borne encephalitis (TBE) is the most significant viral tick-borne zoonotic disease in Europe. The disease is caused by TBE virus (TBEV) infection which often leads to severe encephalitis and myelitis in humans. TBEV is usually transmitted to humans *via* tick bites; however, the virus can also be excreted in the milk of goats, sheep and cattle and infection may then occur *via* consumption of unpasteurised dairy products. Virus prevalence in questing ticks is an unreliable indicator of TBE infection risk as viral RNA is rarely detected even in large sample sizes collected at TBE-endemic areas. Hence, there is a need for robust surveillance techniques to identify emerging TBEV risk areas at early stages.

**Methods:**

Milk and colostrum samples were collected from sheep and goats in Örebro County, Sweden. The milk samples were analysed for the presence of TBEV antibodies by ELISA and validated by western blot in which milk samples were used to detect over-expressed TBEV E-protein in crude cell extracts. Neutralising titers were determined by focus reduction neutralisation test (FRNT). The stability of TBEV in milk and colostrum was studied at different temperatures.

**Results:**

In this study we have developed a novel strategy to identify new TBEV foci. By monitoring TBEV antibodies in milk, we have identified three previously unknown foci in Örebro County which also overlap with areas of TBE infection reported during 2009–2018. In addition, our data indicates that keeping unpasteurised milk at 4 °C will preserve the infectivity of TBEV for several days.

**Conclusions:**

Altogether, we report a non-invasive surveillance technique for revealing risk areas for TBE in Sweden, by detecting TBEV antibodies in sheep milk. This approach is robust and reliable and can accordingly be used to map TBEV “hotspots”. TBEV infectivity in refrigerated milk was preserved, emphasising the importance of pasteurisation (i.e. 72 °C for 15 s) prior to consumption.
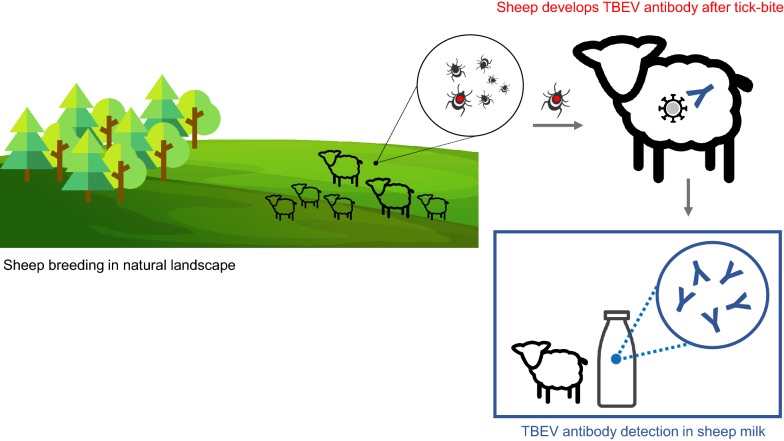

## Background

**Fig. 1:**
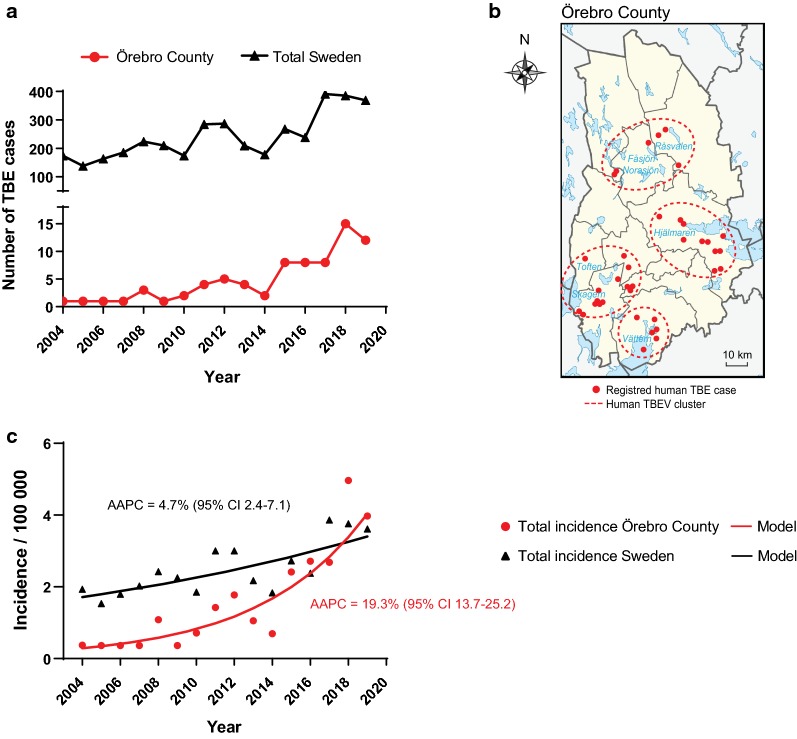
TBE prevalence in Örebro County and Sweden. **a** Number of TBE cases reported from 2004 through 2019 in Örebro County (red) and in all counties in Sweden (black). **b** Reported location of human TBE cases in Örebro County during 2009–2018, indicated by red dots. The cases are confined to four geographical clusters close to lakes: lake Hjälmaren (east); lake Vättern (south); lake Skagern and lake Toften (south-west); and in the northern part of the County where there are many small lakes (Råsvalen, Fåsjön and Norasjön). The clusters are circled in red (dotted lines). **c** Trend analysis of TBE incidence in Örebro County and in Sweden during the period 2004–2019. Joinpoint regression model (Joinpoint trend analysis software [28]) was used to analyse the incidence trends. The average annual percental change (AAPC) was calculated using 95% confidence interval (*P* < 0.05)

Tick-borne encephalitis (TBE) is the most common viral tick-borne zoonosis in Europe and infection may lead to severe central nervous system diseases and result in paralysis [1]. TBE is endemic across Europe and it has been a notifiable disease in the European Union since 2012 [2]. The disease has been registered in Sweden since 1969 by compulsory reporting to the Public Health Agency of Sweden (formerly the Swedish Institute for Infectious Disease Control (SMI)) [3]. Over the last 15 years the number of annual TBE cases in Sweden has increased from 174 in 2004 to 369 in 2019. This increase is seen both in old TBE endemic regions and in new emerging areas. In Sweden, Örebro County is one of these new areas, with no cases reported during the years 2004–2008 and only sporadic cases during 2008–2014, whereas 8–15 cases have been reported annually in 2016–2019 (Fig. 1a). Despite enhanced vaccination coverage, TBE remains a serious threat to Swedish public health. As TBEV is spreading into new areas, the disease is considered a severe emerging threat to humans in Europe and Asia.

Tick-borne encephalitis virus (TBEV) belongs to the family *Flaviviridae*, within the virus genus *Flavivirus*. TBEV has traditionally been divided into three genetically distinguishable subtypes: Western European TBEV transmitted by *Ixodes ricinus*, and Far Eastern and the Siberian TBEV, predominantly transmitted by *I. persulcatus* [4]. In addition, two new subtypes have recently been proposed, these being the Baikalian and the Himalayan subtypes [5, 6]. The virus is maintained in small mammal-tick cycles, whereas human infection is a viral dead end for further transmission [7]. Several factors for the increased prevalence of TBE have been addressed. One reason is climate change influencing tick distribution [8, 9]. The intensity of TBEV transmission depends on the prevalence of infection in the tick population, the density of host-seeking infected ticks and the availability of tick-maintaining hosts [10]. Furthermore, socioeconomic factors and the increasing rate of human outdoor activities also have roles in the expansion of TBE disease in Europe [11].

The geographical distribution of TBE displays a patchy pattern, with each patch representing a TBE focus. Human TBE cases, detection of TBEV in field-collected ticks, in ticks detached from humans, in birds or in animals, as well as in tissues and blood samples from mammals, are all used as golden standards to detect TBEV foci [12]. Since the prevalence of TBEV in the tick population is low and these methods are laborious and involve invasive interventions, there is a need for new and reliable surveillance techniques to identify new TBEV risk areas at an early stage.

TBEV is usually transmitted to humans *via* bites from infected ticks. However, oral transmission from the consumption of unpasteurised dairy products, the so-called alimentary route, has also been reported [13–21]. In these studies, the source of TBEV transmission was primarily either unpasteurised goat milk or cheese. Additionally, a TBE outbreak was reported in Hungary in 2012, where the infection was transmitted by unpasteurised cow’s milk [22]. More recently, TBEV RNA was found in unpasteurised cow milk in Norway [23]. The alimentary route is rare but should be considered as a potential risk factor as TBEV infections have expanded to multiple European sites in recent years. The use of unpasteurised dairy products has received much attention in Sweden and Europe as a whole, as newly established small-scale farms have commenced to produce and sell cheese. There is also an increasing trend of consuming raw food and locally produced self-made products from small producers. The risk of infection depends largely on the stability of the virus in milk and cheese; however, the stability of TBEV in sheep and goat milk at different temperatures has not been thoroughly characterised.

Milk and colostrum (the very first milk produced after giving birth) contain immunoglobulins, which protect the offspring against infections to which the mother has been exposed, and serve as the acquired immunity for the newborn [24, 25]. TBEV has been detected in the milk of goats, sheep and cattle, whereas antibodies against the viral proteins were found only in cattle and sheep milk [15]. Furthermore, milk from domesticated animals offers great advantages for studying the distribution of TBEV in nature, as sheep and goats are confined to smaller areas compared to wild animals, and the sampling does not require invasive methods.

Even though the epidemiology of TBE in Sweden can be described *via* data collections from individual human cases, the use of livestock as sentinels could provide a deeper understanding of how to monitor the disease. In addition, the putative increased risk of unpasteurised milk products that may contain TBEV highlights the importance of further characterising the stability of the virus in milk at different temperatures. In this study, we hypothesise that surveying TBEV-specific antibodies in goat and sheep milk could be a robust way to identify new TBEV foci. Goat and sheep are exposed to ticks and are kept on natural pastures with similar features as natural TBEV foci. We sampled milk from sheep and goat breeds and established a technique to analyse the milk for TBEV-specific antibodies. The technique revealed the presence of previously unknown TBEV foci in the Örebro County, Sweden. Furthermore, we highlight the stability of TBEV in milk at 4 °C.

## Methods

### Human cases

TBE is a notifiable disease in Sweden and all cases that are diagnosed in hospitals and district healthcare centres are reported to the County medical officers (responsible for infectious disease control in the county) in accordance with the Communicable Diseases Act. The cases are then reported and registered by the Public Health Agency of Sweden. All human TBE cases registered in Örebro County during the years 2009–2018 were collected and compiled. Based on interviews with the patients, the cases were mapped to the probable place of infection, i.e. the location where the patient knew or suspected to have been bitten by an infected tick. TBE incidence data was acquired from the Public Health Agency of Sweden for the years 2004–2019 [26].

### Study area

Örebro County covers an area of 8504 km^2^ situated in central Sweden. The population was 303,648 inhabitants in 2019 [27]. The TBE incidence trends for Örebro County and Sweden in total were analysed using the Joinpoint software (Version 4.7.0.0) for joinpoint regression model trend analysis [28] as described previously [29]. Data were acquired from the Public Health Agency Sweden [26].

### Sample collection and preparation

During 2017–2018, all sheep and goat breeders with farms located in Örebro County, Sweden, that were registered with the Swedish Board of Agriculture were asked to participate in the study. Additionally, one sheep farm situated on the island of Blidö in the Stockholm archipelago, Stockholm County, was also asked to participate. Blidö is situated within an area of known risk of TBEV infection. One farm enterprise in the Umeå area, Västerbotten County, was also included. Västerbotten County is a non-endemic TBE region with only three TBE cases reported in the past 10 years [26]. Fifteen milk and 246 colostrum samples were received between 2017–2019 from 37 sheep or goat farms situated in Örebro County, Sweden. As a positive control, milk samples were collected from 4 sheep at a farm situated on the island of Blidö in the Stockholm archipelago. In addition, 14 milk samples from sheep at a farm in the Umeå area, northern Sweden were included as negative controls. Directly after milking an individual animal each milk or colostrum sample was collected into 15 ml plastic test tubes and stored at − 20 °C. These were then stored at the farm until collected by the research team and transported to the laboratory. The samples were thawed on ice and dispensed into 2.5 ml micro tubes (Sarstedt, Nürnbrecht, Germany). Milk fractions were prepared by centrifugation of whole milk at 16,100×*g* for 10 min. A syringe needle was placed beneath the cream layer in the tube and the skim milk was then drawn up by a syringe and transferred to 1.5 ml micro tubes (Sarstedt). Samples of skimmed milk were frozen at − 80 °C until analysed.

### ELISA

The commercially available enzyme-linked immunosorbent assay (ELISA) (Immunozym FSME IgG All Species; Progen Biotechnik GMBH, Heidelberg, Germany) was used for detecting the anti-TBEV-specific antibodies in the milk and colostrum samples. The assay was optimised for milk samples and the samples were analysed according to the manufacturer’s protocol. The optical density was measured at 450 nm (OD_450_) using a Multiskan-Ascent (Lab Systems, Thermo Fisher Scientific, Waltham, MA, USA) spectrophotometer. Using standard curves, sample concentrations were read in Vienna Units per ml (VIEU/ml). Empirical cut-off values (Kunz, Vienna) for TBEV Immunoglobulin G (IgG) antibodies were compared with those based on the Yonden Index and were assessed as follows: negative (< 63 VIEU/ml), borderline (63–126 VIEU/ml) and positive (> 126 VIEU/ml). Samples with borderline or positive titres were further analysed by western blot.

### Cells, transfection and western blot

Baby hamster kidney (BHK-21) cells were maintained at 37 °C and 5% CO_2_ in Dulbecco’s modified Eagle’s medium (DMEM; Sigma-Aldrich, St. Louis, MO, USA) supplemented with 5% foetal bovine serum (FBS) and 1% penicillin and streptomycin (Life Technologies, Carlsbad, CA, USA). BHK-21 cells were grown in T25 culture flasks to reach 70–90% confluence. After trypsinisation, 10^6^ cells were transfected with 4 µg of TBE-ME pCAG plasmid expressing a fused membrane-envelope (ME) protein of TBEV (TBEV-ME-pCAG) using Nucleofector kit L employing a Nucleodector II Device (Lonza, Colonge, Germany) as per manufacturer’s instructions. The transfected cells were incubated at 37 °C and 5% CO_2_ followed by cell lysis with RIPA buffer containing protease inhibitor cocktail (Sigma-Aldrich, St. Louis, MO, USA) at 96 h post-transfection.

Lysate of BHK-21 cells containing TBEV ME protein was separated on sodium dodecyl sulphate polyacrylamide gel electrophoresis gels (NuPAGE 4–12% Bis-Tris gels; Invitrogen, Carlsbad, CA, USA) and transferred to nitrocellulose membranes (iBlot® transfer stacks, nitrocellulose; Thermo Fisher Scientific, Waltham, MA, USA). The membranes were incubated in a blocking solution containing Tris buffered saline with 0.1% Tween 20 (TBS-T) and 5% w/v non-fat dried milk for 1 h at room temperature. After washing three times with TBS-T, the membranes were incubated with the milk samples diluted in TBS-T (1:1) overnight at 4 °C. Following three cycles of washing in TBS-T, the membranes were incubated with a rabbit polyclonal antibody (anti-sheep IgG+IgA+IgM HRP conjugated, LS-C146630; LS Bio, Seattle, WA, USA) diluted in the blocking solution (1:1000) for 1 h at room temperature. For colostrum samples, the membranes were incubated in a blocking solution containing Tris buffered saline with 0.1% Tween 20 (TBS-T) and 10% w/v non-fat dried milk overnight at 4 °C. After three cycles of washing with TBS-T, the colostrum samples were diluted in TBS-T (1:20) and the membranes were incubated with the diluted colostrum overnight at 4 °C. After washing in TBS-T, the membranes were incubated with a rabbit polyclonal antibody (anti-sheep IgG+IgA+IgM HRP conjugated, LS-C146630; LS Bio) diluted in the blocking solution (1:1000) for 1 h at room temperature. The protein bands were visualised using enhanced chemiluminescence substrate (Thermo Fisher Scientific) and ChemiDoc MP Imaging system (BioRad, Hercules, CA, USA). As a loading control for the western blot analysis, the housekeeping protein GAPDH was used. The membranes were re-probed with monoclonal mouse anti-GAPDH antibody (0.05 µg/ml, # G8795; Sigma-Aldrich) overnight at 4 °C. Following three cycles of washing in TBS-T, the membranes were incubated with a secondary polyclonal anti-mouse IgG (H+L) HRP conjugated antibody (Invitrogen) diluted in the blocking solution (1:10,000) for 1 h at room temperature.

### Focus reduction neutralisation test

VeroB4 cells were cultured in medium 199/EBSS (HyClone, GE Healthcare, Chicago, IL, USA) containing 10% FBS, and 1% penicillin and streptomycin (Gibco, Thermo Fischer Scientific). For focus reduction neutralisation test (FRNT), 2-fold serial diluted colostrum was mixed and incubated with 100 focus-forming units (Ffu) of TBEV at 37 °C for 30 min and the mixture was then added to VeroB4 cells for 1 h at 37 °C. After 1 h of incubation, inoculum was removed and foci were revealed by focus forming assay. Neutralising antibody titers was calculated as the reciprocal of the milk dilution that gave 80% reduction of the number of Ffu as compared to virus control.

### Virus stability

100,000 Ffu of TBEV were added per ml of DMEM, milk and colostrum. Virus was incubated for 72 h at 4 °C, room temperature (RT), and at 37 °C. pH and viral titers were measured by means of pH indicator strips (Merck Millipore, Burlington, MA, USA) and focus-forming assay at 0, 24, 48 and 72 h post-inoculation, respectively. TBEV was titrated by means of focus forming assay as previously described [30, 31]. Virus was detected using primary monoclonal mouse antibodies directed against TBEV E (1:1000, 1786 [32]) and secondary anti-mouse HRP-conjugated antibodies (1:2000, Thermo Fisher Scientific). Viral foci were then revealed by incubation with TrueBlue peroxidase substrate (KPL, Gaithersburg, MD, USA). To prevent acidification of the milk due to microbial growth, the DMEM, milk and colostrum were sterilised using beta-propiolactone for 16 h at 4 °C. The beta-propiolactone was then inactivated by incubation at 37 °C for hydrolysis [30].

## Results

### TBE cases

Within the last 10 years, there has been an increase in the number of annually reported TBE cases in Örebro County, Sweden (Fig. 1a). All cases of acquired infection in Örebro County were mapped to the probable place of infection, i.e. the geographical location where the patient considered to have contracted the tick bite (Fig. 1b). The cases were mainly restricted to four different geographical clusters, located close to lakes: lake Hjälmaren in the east; lake Vättern in the south; lakes Skagern and Toften in the south-west; and in an area close to many small lakes in the northern region of the County (Fig. 1b).

A trend analysis of the TBE incidence in Örebro County and all of Sweden within the period of 2004–2019 was performed (Fig. 1c). The incidence of TBE cases in Örebro County increased from 0.37 (in 2004) to 3.97/year/100,000 inhabitants (in 2019). The average annual percentage change (AAPC) was 19.3 (95% confidence interval, CI: 13.7–25.2; *P* < 0.05) during the years 2004–2019. In comparison, the AAPC for total reported TBE cases in Sweden during the same period was 4.7 (95% CI: 2.4–7.1; *P* < 0.05). The incidence of TBEV infection in Sweden consequently increased from 1.93 to 3.61 year/100,000 inhabitants from 2004 to 2019. Since the AAPC is almost four times as high in Örebro County compared to the trend for Sweden as a whole, this highlights Örebro County as an area of emerging TBE disease.

### Establishing a technique for sampling and TBEV antibody detection in milk

**Fig. 2:**
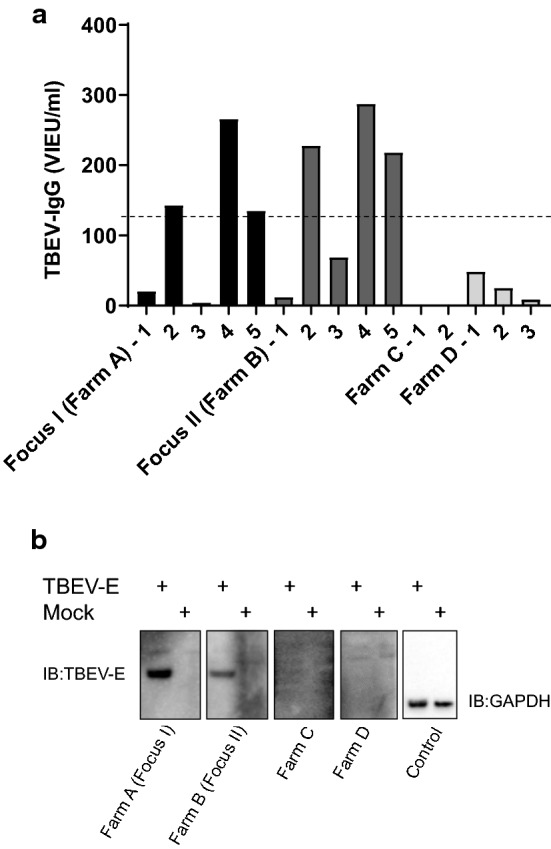
Detecting TBEV antibodies in sheep milk. **a** The milk samples were screened for the presence of anti-TBEV-IgG antibodies using a commercial ELISA (Immunozym FSME IgG All Species; Progen Biotechnik GMBH, Heidelberg, Germany). Antibody titres > 126 VIEU/ml (dotted line) were considered positive, according to manufacturer’s instructions. **b** Western blot analysis of representative milk samples. BHK-21 cells were transfected with plasmid-encoding TBEV membrane and envelope (ME) proteins. Cell lysate was separated on sodium dodecyl sulphate polyacrylamide gel and transferred to nitrocellulose membrane. The milk samples were then used as primary antibody to detect the envelope (E)-protein of TBEV (TBEV-E ≈ 50 kDa). Non-transfected cells are denoted as mock. The expression levels were verified by GAPDH (≈ 35 kDa)

In 2017, 100 sheep and goat breeders in Örebro County, Sweden, that were registered with the Swedish Board of Agriculture, were asked to participate in the study. The response rate was low (4%) and only 15 samples from 4 farms (A–D) were collected in July, 3–4 months post-lambing. The milk samples were screened for the presence of TBEV antibodies using a commercial ELISA (Immunozym FSME IgG All Species, Progen Biotechnik GMBH). In total, 3/5 samples (60%) from farms A and B were positive using the ELISA test, whereas all collected samples from farms C and D were negative (Fig. 2a). To validate the specificity of the method and to further verify the ELISA results, the samples were analysed for the presence of TBEV antibodies by means of a western blot assay. The milk samples were used as the source of primary antibodies to detect the E-protein of TBEV expressed in a crude cell extract. The western blot assay confirmed that TBEV antibodies were detectable in sheep milk from farms A and B, as indicated by an intense band at 50 kDa in the positive samples (Fig. 2b). Altogether, the initial study validated the technique for detecting TBEV foci in milk and revealed two previously unknown TBEV foci, Focus I (Farm A) and Focus II (Farm B) in Örebro County, Sweden.

### Revealing previously unknown TBEV foci in Örebro County by screening sheep and goat colostrum

**Fig. 3:**
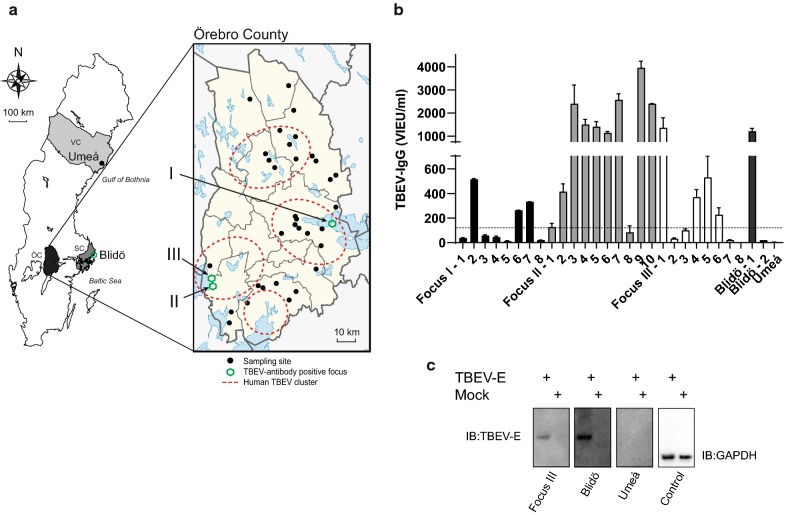
Surveillance and detection of TBEV antibodies by screening of sheep and goat milk, Örebro County, Sweden. **a** Geographical locations of sampling sites and the study area of Örebro County, Sweden. Locations for sample-collection are indicated with red dots. Locations with TBEV antibody-positive milk samples are indicated with green circles, denoted I, II, III and Blidö. The clusters of human TBE cases are circled in red (dotted lines). **b** The milk samples were screened for the presence of anti-TBEV-IgG antibodies using a commercial ELISA (Immunozym FSME IgG All Species; Progen Biotechnik GMBH). Antibody titres > 126 VIEU/ml (dotted line) were considered positive, according to the manufacturer’s instructions. Blidö-1 represents the mean of 3 positive samples. Blidö-2 is a negative sample. Umeå represents the mean titer of 14 samples. **c** Western blot analysis of representative milk samples. BHK-21 cells were transfected with plasmid encoding TBEV membrane and envelope (ME) proteins. Cell lysate was separated on sodium dodecyl sulphate polyacrylamide gel and transferred to nitrocellulose membrane. The milk samples were then used as primary antibody to detect the envelope (E)-protein of TBEV (TBEV-E ≈ 50 kDa). Non-transfected cells are denoted as mock. The expression levels were verified by GAPDH (≈ 35 kDa). *Abbreviations:* SC, Stockholm County; VC, Västerbotten County; ÖC, Örebro County

Based on the feedback from the contacted farmers, we realised that our questionnaires were sent out rather late in the season. Sampling of milk would be easier to access during the lambing and kidding period in the early spring. Breeders also suggested that we should focus on the collection of colostrum (first milk) as it is regularly collected by the farms as back up for lambs and kids not receiving milk properly from their mothers. It could also be an advantage to use colostrum as it contains more antibodies than regular milk. According to this and in order to further expand the study, all sheep and goat breeders in Örebro County, Sweden (approximately 800 farms), were asked to participate in the screening early in 2018. In total, 246 colostrum samples were collected from 5 goat and 30 sheep breeders (4–10 samples from each farm) (Fig. 3a). In addition, samples were collected in an area with reported high levels of TBE (Blidö, an island in the Stockholm archipelago, Stockholm County), and in a non-endemic region (Umeå, Västerbotten County, northern Sweden) (Fig. 3a). In total, 19/264 samples (7.2%) were TBEV antibody-positive using ELISA (Table 1). Colostrum samples collected at Focus I and Focus II were found to be positive (Fig. 3b). Of the 33 additional farms sampled in 2018 in Örebro County, only one breed were found to be positive, thus revealing a previously unknown focus. This was denoted Focus III (Fig. 3b). Focus II is located in close proximity to Focus III, and Foci I-III are all situated within cluster areas for human TBEV cases (Fig. 3a). In the screening of the colostrum samples, 3/8 samples at Focus I, 9/10 samples at Focus II and 4/8 at Focus III were found to be TBEV antibody-positive (Fig. 3b; Table 2). Many of the colostrum samples that were sampled from Focus II revealed an excessively high titer of TBEV antibodies compared to the antibody titers measured at the other two foci. The highest levels are displayed for animals #3 and #9. These sheep are only two years-old, i.e. they were having their first lambs. The farm at Blidö island, a well-known endemic TBEV hotspot, was used as a positive control, and 3 out of 4 samples from Blidö were found to be TBEV antibody-positive (Fig. 3b). Samples collected in the Umeå region were all negative (Fig. 3b). The specificity of the ELISA results was validated by means of the western blot assay, which also demonstrated an intense band at 50 kDa for the colostrum samples from Focus III and Blidö, respectively (Fig. 3c). Taken together, TBEV antibodies were detected in the milk and colostrum at three previously unknown foci located in Örebro County, Sweden. In total, more than 60% of the sampled animals were TBEV antibody-positive at the identified foci, indicating that TBEV present within a pasturage can effectively infect sheep (Table 2).

**Table 1:** Prevalence of tick-borne encephalitis virus antibodies determined by ELISA in colostrum samples from goats or sheep

Animal species	No. of samples	No. of positive samples	% of positive samples	No. of farms	No. of positive farms	% of positive farms
Goats^a^	17	0	0	5	0	0
Sheep	247	19	7.7	30	4	13.3
Total	264	19	7.2	35	4	11.4

^a^All goat milk samples were collected from farms in Örebro County, Sweden

**Table 2:** Determination by ELISA of the prevalence of tick-borne encephalitis virus antibodies in milk and colostrum samples from TBEV positive farms

Animal species	Location	Year	No. of samples	No. of positive samples	% of positive samples
Sheep	Focus I	2017	5	3	60.0
Sheep	Focus II	2017	5	3	60.0
Sheep	Blidö	2018	4	3	75.0
Sheep	Focus III	2018	8	4	50.0
Sheep	Focus I	2019	8	3	37.5
Sheep	Focus II	2019	10	9	90.0
Total			40	25	62.5

**Fig. 4:**
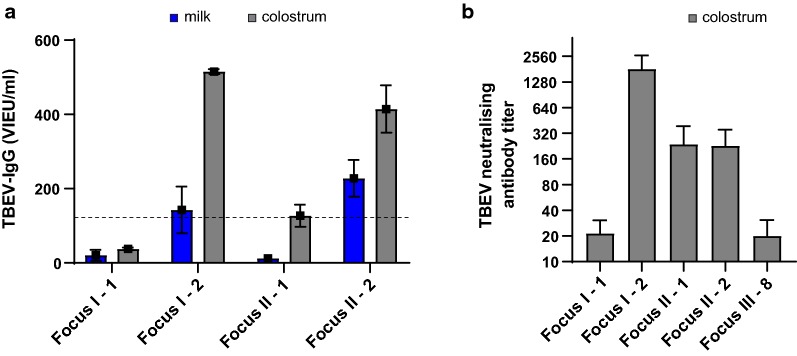
Comparing the presence of TBEV antibodies in milk and colostrum from individual animals over time and neutralisation of TBEV in colostrum. **a** Comparison of anti-TBEV-IgG antibody titer for samples collected from the same individual animals at Focus I (animal #1 and #2) and Focus II (animal #1 and #2) in milk samples (blue) and colostrum samples (grey). **b** Neutralising antibody titers of different colostrum samples detected by means of focus reduction neutralisation test (FRNT). A neutralising titer with colostrum dilution 1/16 and reducing the number of focus forming units (Ffu) by 80% was regarded as positive

Four specific animals were re-sampled for colostrum in 2019, two years after the initial milk-screening at foci I and II. The antibody titer was indeed higher for all the colostrum samples (Fig. 4a). Two of the individual animals initially tested TBEV antibody-positive (animal #2 - Focus I and animal #2 - Focus II) still tested positive with a 2-fold to 4-fold increase. The negative animal #1 at Focus II was now found positive, suggesting that the animal must have encountered TBEV in between the two sampling time points. One animal at Focus I (animal #1) was negative in both samples (Fig. 4a). To analyse the protective neutralising potential of the antibodies present in the milk, FRNT was performed on positive and negative colostrum samples. The positive milk samples from the ELISA assay were able to neutralise the virus, whereas the negative samples did not (Fig. 4b). Altogether, the colostrum tested positive in ELISA and western blot was able to neutralise infectious virus.

### Virus stability

**Fig. 5:**
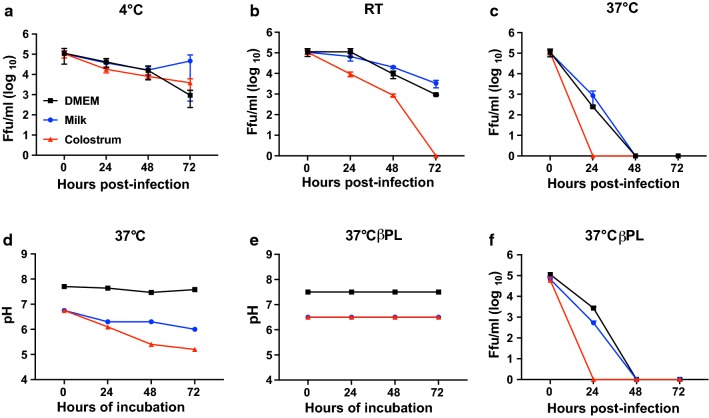
Stability of tick-borne encephalitis virus (TBEV) in sheep milk. TBEV is unstable at room temperature and at 37 °C. 100,000 focus forming units (Ffu) of TBEV were added per ml of DMEM (Dulbecco’s modified Eagle’s medium), milk and colostrum. Virus was incubated for 72 h at 4 °C (**a**), room temperature (RT) (**b**) or 37 °C (**c**), and viral titers were measured by means of focus forming assay, as previously described [15]. DMEM, milk and colostrum were incubated at 37 °C and pH was measured using pH-indicator strips (**d**). DMEM, milk and colostrum were treated with beta-propiolactone for 16 h at 4 °C for sterilisation, followed by incubation at 37 °C for 2 h to hydrolyse and inactivate beta-propiolactone. After sample treatment, 100,000 Ffu of TBEV were added, samples were incubated at 37 °C and pH (**e**) and Ffu (**f**) were measured at the indicated time points

Since TBEV can be transmitted by consuming unpasteurised milk from animals, we were interested in looking at the infectivity of TBEV in milk and colostrum at different temperatures. Milk, colostrum and DMEM were spiked with 10^5^ TBEV Ffu and then incubated at different temperatures. The infectivity of the virus was measured by means of a focus forming assay at different time points. The virus was very stable at 4 °C, compared to room temperature (RT), and 37 °C, whereas complete inhibition was detected after 24 h and 48 h for colostrum and milk respectively (Fig. 5a). Milk is known to become sour when kept at higher temperatures and a low pH is known to modify the virion structure. The change in pH of the milk, colostrum and DMEM over time was determined at different temperatures (Fig. 5b). The pH of the milk and colostrum were only affected at 37 °C. To rule out the effect of pH on virus stability, the milk, colostrum and DMEM were sterilised using beta-propiolactone [30]. Using this treatment, the pH was constant over time at 37 °C in all samples (Fig. 5c). Even though the pH was stable over time, the stability of the TBEV declined rapidly and no infectivity was detected after 24 h and 48 h at 37 °C (Fig. 5d). Taken together, our data indicate that keeping unpasteurised milk at 4 °C will preserve the infectivity of TBEV for at least 3 days, thus stressing the importance of pasteurisation to minimise risk of infection.

## Discussion

An increasing number of human cases of TBE has been recorded in Sweden as TBEV is emerging into new areas and vaccine coverage is incomplete. TBE is emerging into new areas not only in Sweden, but also establishing itself in regions all over Europe and Asia [19, 33, 34]. The epidemiology of TBE in Sweden has changed over the last 20 years. The endemic area has expanded from the area around Stockholm to provinces in the south-west and south of Sweden, regions that are far from previously known endemic areas [8]. A recent study suggests that new TBEV foci have emerged as the density and range of the virus vector *Ixodes ricinus* tick has increased due to the increased availability of the roe deer (*Capreolus capreolus*), thus creating large areas of potential tick habitats in southern Sweden, which in turn is likely to have contributed to the increase of human TBE cases [10]. However, the circulation of the virus in nature and the epidemiology of TBE is dependent on a number of different factors. TBEV is sensitive to various environmental factors, such as the microclimate and the population density of different host animals [8, 10]. A humidity rate of > 85%, air temperatures of > 6 °C to 7 °C, and access to hosts are the vital prerequisites for tick survival [11]. Climate changes with warmer temperatures during winter may also be beneficial to both ticks and hosts, and could thus possibly increase the transmission of the virus [10].

TBEV has a patchy geographical distribution and is restricted to certain areas, often small spots, so-called natural foci [35]. Furthermore, within a given focus the percentage of TBEV-infected ticks is usually as low as < 1% [36, 37]. Therefore, there is no uniform risk of TBE infection in a given limited area as the virus is not uniformly present in a tick population. In order to identify new risk areas and to prevent the disease by vaccination, the locations of emerging TBEV foci need to be identified. Here, a novel robust and reliable technique for monitoring TBEV is presented, which is non-invasive, and which can accordingly be used to map TBEV “hotspots”. By monitoring TBEV antibodies in milk, three novel foci in Örebro County were identified, which also overlap with the plausible place of infection of registered human TBE cases reported during 2009–2018. During the grazing season, sheep and goats breed in rural pasturage, areas that are well-known tick habitats. Furthermore, the grazing season also overlaps with the months of onset when most TBE cases are reported, i.e. May to October [8, 33].

The use of livestock as sentinel animals has been proposed as a target strategy for assessing the risk of TBEV infection in regions where TBE prevalence is low [12, 38]. In a recent study, goats have been used as sentinel animals in order to identify new risk areas of TBE in south-west Switzerland [39]. Goat sera were screened for TBEV antibodies, and at two of the three locations where goats were seropositive, the local tick populations also tested positive for TBEV. In a recent study, sera from goats and sheep were analysed in north-eastern Germany. None of the tested sera were positive, but 11/479 (2.3%) sera were tested borderline for anti-TBEV-IgG using ELISA. However, two sheep sera tested positive using a virus neutralisation test [40]. The presence of TBEV in goat and human sera and field-collected ticks was investigated by Casati Pagani et al. [41]. Seroprevalence in the goat sera was 14.6%, but in the human sera, from goat owners in that region, there were no detectable TBEV antibodies. Sheep have also been used for identification of TBE risk areas in north-western Romania [42]. Serum from adult sheep was collected in five counties and TBEV was identified using virus neutralization test in 15.02% of sheep tested. Positive samples were found in 40 of 50 examined locations.

In Sweden, there are more sheep than goats registered (600,000 compared to 20,000) [43, 44], reflecting the cultural tradition of breeding animals in Sweden. The distribution of sheep and lamb livestock covers almost all regions in Sweden, with the southern (Götaland) and central (Svealand) regions representing almost 90%. Within the region of northern Sweden (Norrland), herd density is low, comprising only 10% of the total livestock, which reflects both the natural landscape with its temperate climate and mountainous landscape as well as the demographics of northern Sweden. Swedish sheep livestock-keeping also supports large, open grazing lands and natural pastures, rather than closed pasturage [45]. During the summer, sheep are also kept in areas used by people for recreational activities, such as hiking paths in nature reserves. Based on results from this study, sheep are suggested to be a more representative sentinel species than cattle. Dairy cattle are usually kept in grassland paddocks on arable land close to the stables. Moreover, sheep are susceptible to ticks, and different tick-borne pathogens including *Anaplasma phagocytophilum* have recently been reported in lambs at Swedish farms [46]. Also, sheep have relatively short legs, soft woollen fur, and often inhabit high grass pasturage areas, in comparison to cattle with longer legs and a different type of fur. This was also confirmed when interviewing the farmers at the foci, who demonstrated where ticks often stick to the sheep body, namely at moist locations on the neck, the stomach, the legs and the udder. For epidemiological screening of TBEV, we believe that milk from sheep is promising as a sentinel, particularly as sheep graze in small enclosed pasturage areas of limited size. The pasturage is used from year to year and there is very limited movement between different areas. In a given endemic area, the sheep might be infected by infected ticks and then develop TBEV antibodies.

Sampling of milk is a non-invasive technique that requires no veterinary surgeons, as would be the case for the sampling of sera, as described by Rielle et al. [39]. The colostrum is only available for collection at the time of birth of the lamb [47] and can preferably be collected by the farmer, since farmers routinely collect the first milk for feeding and storage in case a lamb needs assistance [48]. After giving birth, the ewe tends to be very protective and it is difficult to get access to the milk when the sheep and lambs are on pasturage and not kept in a stable. Our data suggest that we can detect TBEV antibodies in milk regardless of the time of sampling post-birth. The concentration of IgG in sheep milk has been found to be at its highest level at the time of birth of the lamb, followed by a post-partum decline [24]. In our study, TBEV antibodies in the milk from the same animal could still be detected after 2 years. However, we cannot rule out reinfection during this time period. This correlates with data reported from Klaus et al. [49] where the persistence of TBEV antibodies could be demonstrated after 28 months. The present study was restricted to areas of pasturage where sheep and goats breed, which restricted the study area. This study also relied on volunteer sampling and consent for participation, which further determines which farms and thereby areas can be screened.

The stability of Langat virus (LGTV), a BSL-2 model for TBEV, has been studied in goat milk [50]. When kept at refrigerated temperatures for several days, LGTV was stable. Once the virus was incubated in goat milk at room temperature, the virus was present for 24 h but was undetectable after 48 h. Furthermore, it was also demonstrated that the virus was completely inactivated after a so-called high temperature short time pasteurisation treatment (72 °C for 15 s) [50]. We show here that sheep milk and colostrum infected with TBEV preserves the infectivity of the virus for several days if kept at 4 °C. Complete inhibition was detected after 24 h and 48 h for both colostrum and milk when kept at 37 °C. To prevent further outbreaks of alimentary TBEV infections the milk should be pasteurised before consumption. This study further indicates the importance of promoting pasteurisation due to the increasing trend of consuming raw dairy product. We were also able to show that sheep previously infected with TBEV with antibodies in their milk efficiently neutralise TBEV.

In this study, we have developed a novel strategy for identifying new TBEV foci. By monitoring TBEV antibodies in sheep and goat milk we have identified three novel foci in Örebro County, Sweden, which also overlaps with areas of TBE infection reported during the years 2009–2018. Further studies with a broader geographical coverage and greater sample size should be carried out in order to further address the prevalence of TBEV antibodies in livestock milk. A monthly sampling and year-to-year follow-up of a greater number of individual animals is also of interest in order to further investigate and monitor how TBEV antibody titers in colostrum and milk vary with time, season and age of the animal. We also highlight that infectious TBEV could be present for several days in sheep milk and colostrum kept at 4 °C.

## Conclusions

Our data show the presence of TBEV antibodies in sheep milk from Swedish farms, indicating the presence of TBEV at novel foci located in Örebro County. We suggest sheep milk as a suitable sentinel in order to identify novel TBEV foci. As TBE spreads into new areas, this technique has great potential as a novel surveillance strategy for the future monitoring of the virus. Furthermore, it is of considerable importance to promote the pasteurisation of dairy products in order to minimise the risk of alimentary TBEV outbreaks.

## Data Availability

Data supporting the conclusions of this article are included within the article. Human TBE incidence data are available at https://www.folkhalsomyndigheten.se/folkhalsorapportering-statistik/statistik-a-o/sjukdomsstatistik/tick-borne-encephalitis-tbe/?t=county. Population statistics are available at https://www.scb.se/hitta-statistik/statistik-efter-amne/befolkning/befolkningens-sammansattning/befolkningsstatistik/pong/tabell-och-diagram/kvartals–och-halvarsstatistik–kommun-lan-och-riket/kvartal-1-2019/.

## Abbreviations

AAPC: average annual percent change
ELISA: enzyme-linked immunosorbent assay
Ffu: focus forming units
FRNT: focus reduction neutralisation test
IgG: immunoglobin G
E: envelope protein
ME: membrane-envelope protein
RNA: ribonucleic acid
TBE: tick-borne encephalitis
TBEV: tick-borne encephalitis virus
VIEU: Vienna Units
